# The Diversity and Evolution of Sex Chromosomes in Frogs

**DOI:** 10.3390/genes12040483

**Published:** 2021-03-26

**Authors:** Wen-Juan Ma, Paris Veltsos

**Affiliations:** 1 Department of Molecular Biosciences, University of Kansas, Lawrence, KS 66045, USA; 2 Department of Ecology & Evolutionary Biology, University of Kansas, Lawrence, KS 66045, USA; parisveltsos@gmail.com

**Keywords:** anurans, sex chromosome diversity, homomorphy, heteromorphy, sex determination, fountain of youth, sexually antagonistic selection, sex-determination turnover, telomere-restricted recombination

## Abstract

Frogs are ideal organisms for studying sex chromosome evolution because of their diversity in sex chromosome differentiation and sex-determination systems. We review 222 anuran frogs, spanning ~220 Myr of divergence, with characterized sex chromosomes, and discuss their evolution, phylogenetic distribution and transitions between homomorphic and heteromorphic states, as well as between sex-determination systems. Most (~75%) anurans have homomorphic sex chromosomes, with XY systems being three times more common than ZW systems. Most remaining anurans (~25%) have heteromorphic sex chromosomes, with XY and ZW systems almost equally represented. There are Y-autosome fusions in 11 species, and no W-/Z-/X-autosome fusions are known. The phylogeny represents at least 19 transitions between sex-determination systems and at least 16 cases of independent evolution of heteromorphic sex chromosomes from homomorphy, the likely ancestral state. Five lineages mostly have heteromorphic sex chromosomes, which might have evolved due to demographic and sexual selection attributes of those lineages. Males do not recombine over most of their genome, regardless of which is the heterogametic sex. Nevertheless, telomere-restricted recombination between ZW chromosomes has evolved at least once. More comparative genomic studies are needed to understand the evolutionary trajectories of sex chromosomes among frog lineages, especially in the ZW systems.

## 1. Sex Chromosome Evolution

Across the tree of life, species determine sex either by using environmental cues or with sex chromosomes, which are the subject of this review. The asymmetrical inheritance of sex chromosomes with respect to sex makes them a genomic hotspot, compared to autosomes, for sex-specific selection and sexual conflict [1,2,3,4]. Sex chromosomes have independently evolved multiple times and show varied levels of divergence from each other in the heterogametic sex (in XY males or ZW females; [5,6,7,8,9,10]). The Y/W chromosomes in mammals, most birds and insects, are highly differentiated, and many functional genes have degenerated or were lost completely due to the long-term arrest of recombination with the X/Z. In sharp contrast, sex chromosomes are usually homomorphic (indistinguishable under the microscope) and rich in gene content in many reptiles, fish, amphibians and dioecious flowering plants and are thought to represent early stages of sex chromosome evolution [7,10,11,12,13]. The reason why the trajectory of sex chromosome evolution differs so dramatically across the tree of life is an unresolved question in evolutionary biology.

The canonical model of sex chromosome evolution suggests that sexually antagonistic (SA) genes play a key role in the process of sex chromosome degeneration [5,14,15,16,17,18]. Sex chromosomes are thought to evolve from a pair of autosomes starting with a sex-determining mutation. Male beneficial mutations are favored to accumulate in the vicinity of the sex-determining locus on the sex-limited Y chromosome (or female-beneficial mutations on the W), which selects for the arrest of recombination in the region because it contains the fittest possible haplotype for the heterogametic sex. The arrival of new mutations with sex-specific beneficial effects selects for an extension of the region of recombination arrest to a longer haplotype. Over evolutionary time, the recombination arrest leads to progressive differentiation and degeneration of sex-limited chromosomes through mechanisms, such as Muller’s ratchet, genetic hitchhiking, reduced purifying selection, and stronger genetic drift [5,14,15,16,17,18]. This model has been widely accepted to account for the recombination suppression and degeneration in the highly differentiated sex chromosomes in mammals, most birds and insects [7,10].

In the past decade, with the advancement and reduced price of genomic sequencing, studies of many non-model organisms have revealed a remarkable diversity in the rate of sex chromosome differentiation, as well as in the dynamics of birth and death of sex chromosomes (i.e., sex chromosome turnovers) in many fishes, amphibians and reptiles (reviewed by [19]). In many cases, there is very limited differentiation between the sex chromosomes in the heterogametic sex despite their old age [20,21,22,23,24,25]. In other words, they do not follow the degeneration path predicted by the canonical model of sex chromosome evolution. Furthermore, there has been little empirical support to demonstrate the role of sexually antagonistic (SA) genes in sex chromosome recombination suppression, because the phenotypic effects of SA genes are difficult to demonstrate and because it is hard to show the causal effects of SA genes (since SA genes precede recombination arrest, both recombining and arrest status are needed in closely related species to test this; reviewed by [4]). In some frog lineages, studies have shown that recombination suppression between XY chromosomes is likely due to genome-wide male-specific reduced recombination, because it is restricted to the telomeric regions in males (females recombine across the full length of their chromosomes), which challenges the universal role of sexually antagonistic (SA) genes in driving sex chromosome recombination arrest [26,27,28,29]. Studies on a variety of stages of sex chromosome evolution from homomorphic to highly degenerated, particularly the less studied early stage, are still needed to fully understand sex chromosome evolution.

## 2. Sex Chromosome Diversity in Frogs

Frogs offer an ideal system for advancing our understanding of sex chromosome diversity and evolution; this is because they harbor various stages of sex chromosome differentiation and diverse sex-determination systems between different species and, sometimes, across and within populations of the same species [26,30,31,32,33]. Earlier studies showed that the majority (~95%) of studied frog species had homomorphic sex chromosomes [30,33]. Recent studies have revealed a diversity of sex chromosome systems in frogs, including heteromorphic and multiple sex chromosomes, especially in species distributed in neotropical regions (i.e., Central and South America) [12,34,35,36]. Heteromorphic sex chromosomes show signs of degeneration, such as extensive accumulation of transposable elements and other repeats, resulting in an enlargement of the sex-limited chromosomes (Y or W), increased heterochromatinization, or a diminishment of their size and gene loss. Both are consequences of the long-term recombination suppression between the sex chromosomes [13,37].

**Figure 1:**
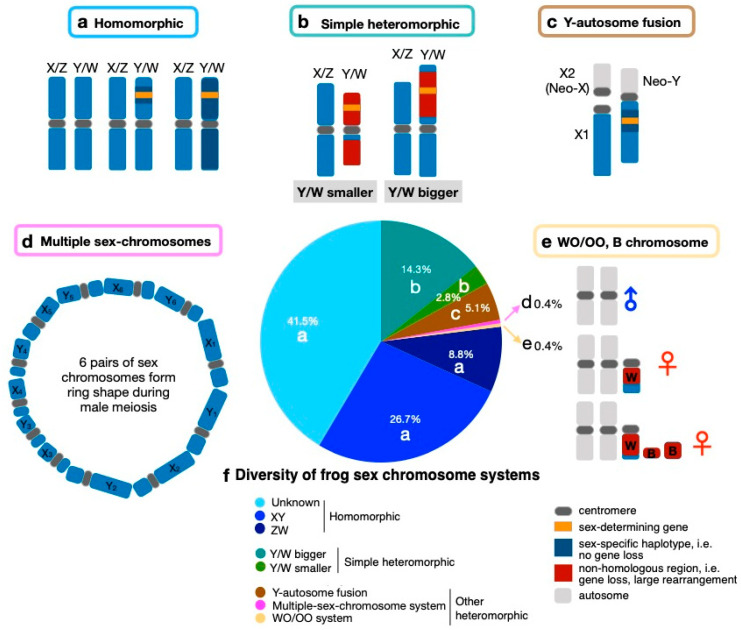
Diversity of frog sex chromosome systems. (**a**) Homomorphic sex chromosomes—sex-specific haplotype can vary largely from very small to the full length of the homologous X/Z, within or across populations within species. (**b**) Simple heteromorphic sex chromosomes—the Y/W may be bigger or smaller than X/Z. (**c**) Y-autosome fusion generating Neo-X (X_2_) and Neo-Y. (**d**) Multiple sex chromosomes in *Leptodactylus pentadactylus*, where 6 pairs of sex chromosomes form a ring shape during meiosis. (**e**) WO/OO and supernumerary (B) chromosomes in *Leiopelma hochstetteri*, with population-specific W and various forms of B chromosomes, which are never observed in males. (**f**) Pie chart shows the proportion of sex chromosome systems in Anuran frogs. All the non-homomorphic sex chromosome types, such as b, c, d and e systems, are heteromorphic.

In this review, we have compiled a list of 222 Anuran species, from 23 different families, with known sex chromosome systems from the literature, spanning ~220 Myr (Supplementary Materials Table S1). We discuss the pattern, diversity and evolution of their sex chromosomes. The majority (~75%) have homomorphic sex chromosomes (Figure 1a, Table S1), with more male heterogametic (26.7%, XX/XY) than female heterogametic (8.8%, ZW/ZZ) systems, and 41.5% with an unknown system (Figure 1f). The DNA sequence difference between two sex chromosomes varies from most of their length to being restricted to the sex-determining region, or no differentiation, as detected in populations of the common frog *Rana temporaria* (Figure 1a, [38,39]). Less commonly, overall, ~25% of frog species have heteromorphic sex chromosomes (Figure 1f). Among the simple heteromorphic sex chromosome system (composed of a single pair), there are slightly more ZW (N_species_ = 23–24) than XY (N_species_ = 19–20, *Glandirana rugosa* has both XY and ZW) systems (Figure 1b). The second commonest heteromorphic sex chromosome system involves Y-autosome fusions (no fusion with a W is known), which occur in ~5.1% of all surveyed species (Figure 1c). One interesting sex chromosome system was discovered in the Brazilian smoky jungle frog (*Leptodactylus pentadactylus*) which has six pairs of sex chromosomes and five autosomal pairs [40] (Figure 1d). Another unique sex chromosome system involves the coexistence of female-specific W (WO/OO system) and supernumerary (or B) chromosomes of various sizes among populations in the New Zealand frog *Leiophlma hochstetteri* [41,42] (Figure 1e).

**Figure 2:**
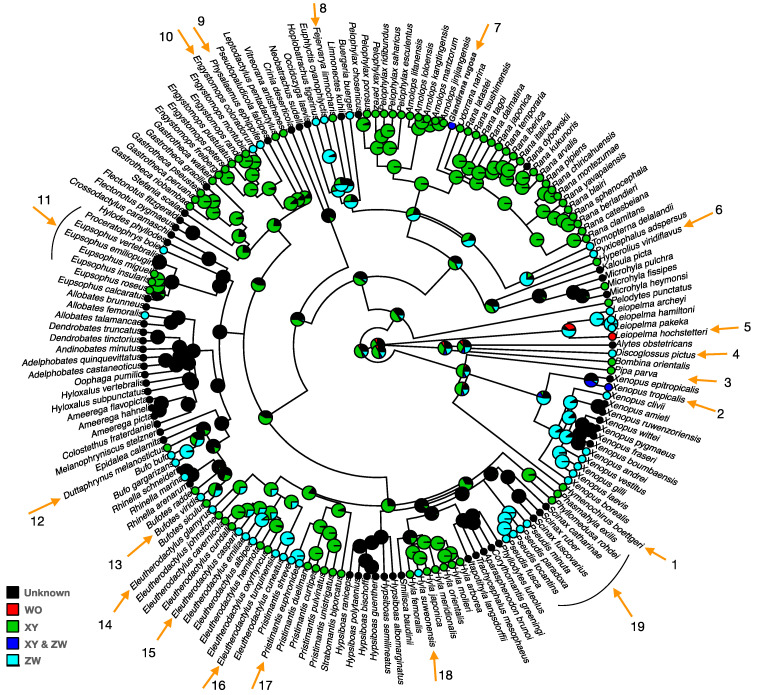
Distribution of various sex-determination systems along Anurans. The phylogenetic tree is obtained from http://www.timetree.org/, accessed on 5 February 2021 and the pattern of sex chromosome diversity and the ancestral status reconstruction were analyzed and visualized with the “Phytools” package in R (version 3.6.3), using a stochastic mapping approach with the ARD (all rates different matrix) transition rate model for discrete character traits [48,49]. Pie charts display the posterior probability of sex-determination systems on each node of the phylogenetic tree. Numbered arrows indicate transitions between sex-determination systems.

There are at least 19 turnovers between sex-determination systems (including XY to ZW, ZW to XY, and ZW to WO) during anuran evolution spanning ~220 Myr (Figure 2, Figure S1). It was not possible to reconstruct the ancestral status due to many transitions along the phylogeny (Figure 2), but the stochastic mapping approach using the ARD model estimated XY had the highest probability among the known sex-determination systems. The sex-determination system is almost exclusively XY in Ranidae frogs (with the exception of *Glandirana rugosa* which has both XY and ZW systems) and in *Hyla* tree frogs (except *H. suweonensis* which has ZW). All *Pseudis* frogs have a ZW system. The number of sex-chromosome transitions is expected to be higher than the number estimated for sex-determination system transitions (Figure 2), because turnovers between chromosomes within the same sex chromosome system are common. There are at least 13 turnovers within almost exclusively the XY system in 28 Ranidae frogs [43]. Frequent sex-determination system turnovers also occur in geckos (17–25 transitions [44]), stickleback fishes (2–3 transitions, [45]) and salmonid fishes (3 transitions, [46]). Sex chromosome turnover within a given sex-determination system has also been documented in *Oryzias* medaka fishes (five transitions, [47]).

## 3. Sex Determination in Frogs

**Figure 3:**
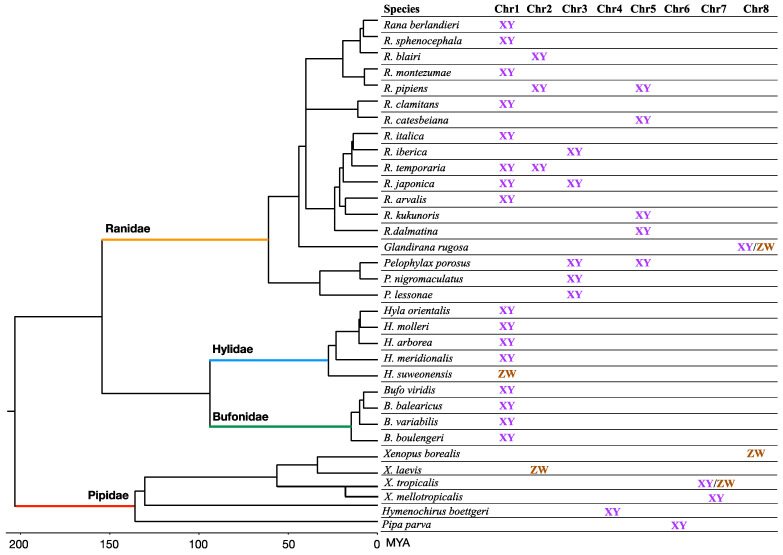
Overview of known sex-determination systems among anurans across four families (branch in distinct colors) spanning ~200 Mya divergence, due to the well-conserved karyotype and genome-wide synteny revealed by comparative genomics. The chromosome number is based on the genome of *Xenopus tropicalis*. The chromosomes (Chr9 and Chr10) that are never used as sex chromosomes are omitted. Sex chromosomes were identified from various publications [43,55,59,60,62,63,64,65,66,67]. The tree is obtained from http://www.timetree.org/, accessed on 5 February 2021 and visualized in Figtree (http://tree.bio.ed.ac.uk/software/figtree/, accessed on 5 February 2021).

Sex in most amphibians is genetically determined, although non-genetic sex determination has also been reported [50]. Many genes with a known association with gonadal differentiation have been mapped to the sex chromosomes of various frog species. Genes with feminization effects include *Dm-w*, *Cyp19*, *Sf1*, *Foxl2*, *Sox3*, and genes with masculinization effects include *Dmrt1*, *Amh*, *Ar*, *Cyp17* (reviewed in [32,51]). Little is known about the molecular mechanisms underlying sex determination in frogs. The only confirmed sex-determining gene is *Dm-w*, located on the W chromosome of the African clawed frog *Xenopus laevis*, where it has a crucial role in primary ovary formation [52,53]. *Dm-w* shares high protein sequence identity (~89%) with the DNA-binding domain of its paralog *Dmrt1*, but does not contain a domain with homology to the transactivation domain of *Dmrt1* [54]. It has been hypothesized that *Dm-w* binds the target gene *Dmrt1* in the gonads of ZW females during sex determination, which prevents *Dmrt1* from interacting with its binding site, thus inducing ovary formation [52,53]. A recent study has found that the *Dm-w* gene is not always associated with female development in many Pipidae species (except in *Xenopus laevis*, *X. gilli*) and has further identified sex chromosomes in three additional pipid frogs (see Figure 3, [55]). 

Frog genomes exhibit considerable synteny [56,57,58], despite long periods of independent evolution (e.g., approximately 266 Myr of divergence between *Narorana parkeri* and *Xenopus tropicalis* or 204 Myr between *Rana temporaria* and *Xenopus tropicalis*). Genome-wide syntenic block analysis shows that amphibians have fewer inter-chromosomal rearrangements than mammals but have a comparable rate of intrachromosomal rearrangements [56]. It is therefore possible to refer to the chromosome numbers of *Xenopus* when comparing frog genomes (Figure 3).

A study in three divergent groups of anurans (*Bufo siculus*, *Hyla arborea* and *Rana temporaria*) found that various sex-linked genes, including *FGA*, *SMARCB1*, *Dmrt1*, map to *Xenopus tropicalis* chromosome 1 (Chr1) and show a strong association with sex (Figure 3, [58]). Other studies using microsatellites found sex-linked haplotypes on Chr1, in addition, in at least four *Hyla* tree frog species and four *Bufo* species [59,60]. *Dmrt1* is known to play a key role in sex differentiation across many animal lineages. Advances in RAD (restriction site associated DNA) sequencing allow us to rapidly expand the identification of sex chromosomes in many more species. A recent study identified five chromosomes (Chr1, Chr2, Chr3, Chr5, Chr8) in 28 Ranidae to be associated with sex determination (Figure 3) [43]. The non-random co-option of these five chromosomes as sex chromosomes was probably because genes on these chromosomes are involved in the sex-determination cascade in amphibians. Such candidate genes include *Dmrt1* and *Amh* on Chr1, *Fgf9*, *Amhr2* and *Rspo1* on Chr2, *Cyp19* on Chr3, *Foxl2* on Chr5, *Sox9* on Chr7, *Ar* and *Sox3* on Chr8 [43,51,61]. Furthermore, one study has further extended the sex chromosome list in pipid frogs (Chr2, Chr4, Chr6, Chr7, Chr8) [55], which leaves homologs of only *X. tropicalis* Chr9 and Chr10 as not being associated with sex in frogs.

## 4. Homomorphic Sex Chromosomes in Frogs

**Figure 4:**
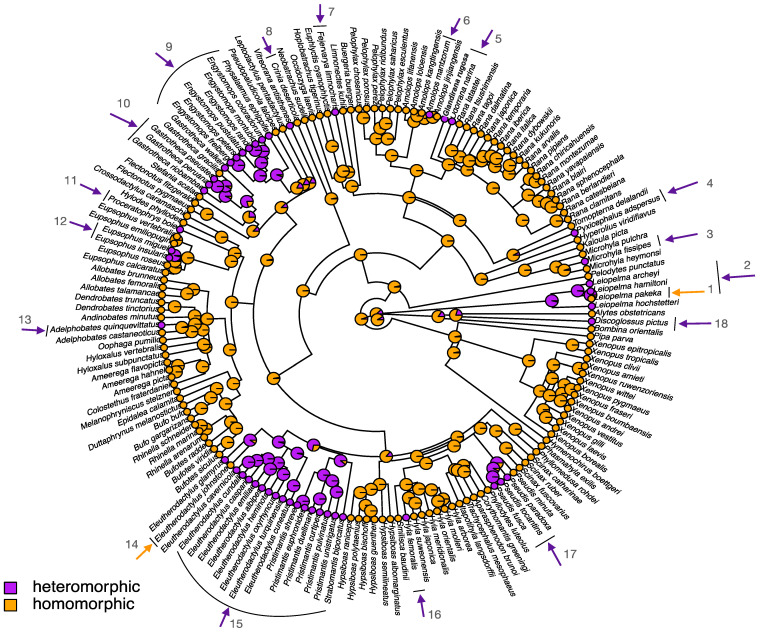
Distribution of homomorphic and heteromorphic sex chromosomes along the phylogeny of studied anurans; see more details in Table S1. Homomorphic sex chromosomes are indicated in orange, and heteromorphic in purple. The tree is obtained from http://www.timetree.org/, accessed on 5 February 2021 and the ancestral state reconstruction of sex chromosome morphology is analyzed and visualized with the “Phytools” package in R (version 3.6.3), using a stochastic mapping approach with the ER (equal rate) transition rate model, suitable for binary character traits [48,49]. Pie charts display the posterior probabilities of sex chromosome types on each node of the phylogenetic tree. Numbered arrows indicate transitions in sex chromosome morphology, purple arrows indicate from homomorphy to heteromorphy, and orange arrows from heteromorphy to homomorphy.

The majority (~75%) of studied anurans (20/23 studied families) have homomorphic sex chromosomes (Figure 1, Table S1). Most species with homomorphic sex chromosomes occur in Bufonidae (100% of the family), Pipidae (100%), Dendrobatidae (91%), Ranidae (88%) and Hylidae (88%) (Figure 4). Among species with homomorphic sex chromosomes, there are three times more species (N_species_ = 57–58) with male heterogamety (XX/XY), than those (N_species_ = 18–19) with female heterogamety (ZZ/ZW). The remaining 54% of species with homomorphic sex chromosomes have an unknown (NA) sex chromosome system due to limited cytogenetic analysis (Table S1).

One of the best studied homomorphic sex chromosome systems is in the European common frog *Rana temporaria*. This species is distributed in Europe as far north as Scandinavia and as far east as the Urals, but not in most of Iberia, southern Italy and the southern Balkans [68,69,70]. Consistent with earlier karyotype studies [71], genetic and genomic studies have confirmed the species to have a homomorphic male heterogametic XX/XY system [38,72,73]. Variation in the *Dmrt1* gene is consistent with a sex-determination role across populations in this species [38]. There is an interesting polymorphism in Y-chromosome differentiation (Chr1), which was identified using sex-linked markers, including the *Dmrt1* region. The Y-chromosome may be fully differentiated (Y-specific haplotypes as long as Chr1), proto-Y (Y-specific haplotype is restricted to the region surrounding *Dmrt1*), and undifferentiated Y (no Y-specific haplotype is identified) (Figure 1a, [38,39,73]). Multiple haplotypes of each Y-chromosome differentiation pattern have been found [39,74]. The three levels of Y-chromosome differentiation follow a latitudinal cline within Sweden, where populations in the south have the majority (>81%) of males with proto-Y. This proportion decreases as latitude increases so that northern Swedish populations exclusively have a fully differentiated Y-chromosome haplotype [73]. The association with temperature, implied by this latitudinal cline, is not repeated in Swiss populations where temperature varies with altitude. Instead, Y-chromosome haplotypes are better associated with the phylogeographic signal, similar to the distribution pattern of mitochondrial haplotypes [74,75]. Remarkably, Y-chromosome haplotype polymorphisms involving all three differentiation levels were found within single Swiss populations [39,72], and the haplotypes appear selectively neutral [29]. The forces maintaining the polymorphism within populations remain unclear [29,39].

Intraspecific polymorphisms of homomorphic sex chromosomes have been documented in many frog species (Figure 3). Chr1 is the sex chromosome in *R. temporaria* throughout its distribution range, but Chr2 has also been shown to co-segregate with sex (along with Chr1) in one northern Swedish population [66,67]. *Pelophylax porosus* uses Chr5 in the Okayama form in western Japan and Chr3 in the Nagoya form in eastern Japan [76]. Two chromosomes in *Rana pipiens*, Chr2 and Chr5, have been described as sex-linked in various populations between lineages of eastern and western USA [43,77]. *R. japonica* uses Chr1 to determine the sex in western Japan but Chr3 in eastern Japan [78]. However, no association was found between sex and any of these two chromosomes in the Akita (northern Honshu) population, suggesting another undetected intraspecific sex chromosome polymorphism in *R. japonica* [78]. Chr8 is probably involved in the intraspecific sex-determination system turnover in *Glandirana rugosa*, from an XY to ZW sex-determination system [61]. Finally, *Xenopus tropicalis* Chr7 is associated with multiple sex-determination system turnovers, where W, Z, and Y chromosomes occur in the same population in Ghana, and the degenerate W and Y probably evolved from the Z chromosomes [62].

### 4.1. Mechanism/Forces to Maintain Homomorphic Sex Chromosomes

Two mutually nonexclusive hypotheses have been proposed to explain the maintenance of homomorphic sex chromosomes in frogs. The “fountain of youth” model states that deleterious mutations could be purged from the sex-limited chromosomes (Y or W), and long-term differentiation could be prevented by occasional sex-chromosome recombination [79]. Supporting evidence has been found in European tree frogs, which inherited the same pair of sex chromosomes from a common ancestor approximately 5 Mya ago. Their sex chromosomes remained homomorphic despite the lack of XY recombination in males, and haplotypes at sex-linked markers further cluster by species and not by gametologs, suggesting a history of recurrent XY recombination [20,22,80]. Using an Approximate Bayesian Computation approach, Guerrero et al. [21] showed the rate of XY recombination in this tree frog group was significantly different from zero, while being ∼10^−6^ lower than that of XX recombination. A possible mechanism allowing XY recombination is the occasional sex reversal of XY individuals (i.e., sex-reversed XY females), resulting from incomplete genetic control over sex determination [79]. If the arrest of XY recombination in males is a property of male meiosis and not the male genotype, then Y chromosomes would recombine with X chromosomes in the occasional sex-reversed XY females, preventing Y chromosomes from progressive differentiation and degeneration. Sex-reversal experiments in a series of taxa (crested newts, medaka fish, housefly) have confirmed that sex differences in recombination largely depend on phenotypic sex, not genotypic sex [81,82,83]. Additional support for this “fountain-of-youth” model comes from genetic mapping in wild populations of the common frog *R. temporaria*, which showed that XY recombination only depends on phenotypic sex. Wild XX males showed recombination restriction similar to XY males, while wild sex-reversed XY females recombined as much as XX females [84].

The second hypothesis for maintenance of homomorphic sex chromosomes states that sex chromosome turnovers are frequent enough that sex chromosomes are replaced by another autosome pair before they have time to degenerate [85,86]. Van Doorn and Kirkpatrick [87,88] proposed a role for sexually antagonistic (SA) genes in driving sex chromosome turnover, where a male-benefiting mutation accumulating on an autosome favors the evolution of a masculinizing mutation in its vicinity. This mechanism is well-illustrated in Cichlidae fishes but with a female-beneficial mutation, which allowed a new ZW system to invade an XY system via a mutation on the proto-W chromosome [89]. Blaser et al. [90] proposed a role for the mutation load that accumulates on sex chromosomes to drive the sex chromosome turnovers. Later, Blaser et al. [91] proposed the “hot-potato model”, which combined SA and deleterious effects. They showed that SA alleles located on a chromosome, after it has been co-opted for sex, induce the recombination arrest, and the ensuing accumulation of deleterious mutations would generate pressure for a new sex-chromosome transition. This “hot-potato model” makes two predictions, (1) that the type of heterogamety would be conserved during transitions, and (2) that autosomes are not recruited randomly, with some autosomes being more likely to be co-opted for sex. Both predictions were supported in a study of 28 Ranidea, which showed at least 13 sex chromosome turnovers while preserving (with one exception) the XY system, although genetic drift could not be excluded to account for fast sex chromosome turnovers. Similarly, the model is supported by the frequent sex chromosome turnovers within *Oryzias* medaka fishes, where five turnovers occur within the XY system and two in the ZW system [47].

## 5. Heteromorphic Sex Chromosomes in Frogs

Recent frog studies have brought up the proportion of frog species with heteromorphic sex chromosomes to ~25% among these karyotyped frogs (Table S1). Among the simple heteromorphic sex chromosomes, female heterogametic (ZW) species are slightly more common than those with male heterogamety (XY, N_species_ = 23 vs. 19, *Glandirana rugosa* has both XY and ZW systems). Interestingly, 68% of these species with XY systems and 83% with ZW systems have larger Ys/Ws chromosomes than their homologous Xs/Zs chromosomes (Table S1, Figure 1b), suggesting most of the simple heteromorphic sex chromosome systems in frogs are at a relatively early stage of sex chromosome evolution.

Heteromorphic sex chromosomes in frogs have been relatively understudied, and the forces allowing their escape from the mechanisms maintaining homomorphy in most frog species remain largely unknown. Phylogenetic analysis suggests the likely ancestral status to be homomorphic sex chromosomes in frogs (88% probability; Figure 4). Heteromorphic sex chromosomes span several lineages and also occur within closely related species in genera with primarily homomorphic sex chromosomes, suggesting that heteromorphy has independently evolved multiple times (at least 16; Figure 4). Interestingly, the inverse transition (from heteromorphy to homomorphy) is rare (two times in our compiled dataset; Figure 4). Five frog genera have primarily heteromorphic sex chromosomes (*Eleutherodactylus*, *Gastrotheca*, *Pristimantis*, *Engystomops* and *Pseudis*; Figure 4). The stability of the sex-determination system varies between the lineages, with *Pseudis* being exclusively ZW, *Pristimantis* and *Gastrotheca* having mostly a XY system (60–80% of species), and *Eleutherodactylus* has an equal proportion of XY and ZW systems (Figure 2).

The canonical sex chromosome evolution model predicts that the Y and W chromosomes eventually become small because they degenerate and that most of their genetic content is lost due to the absence of recombination with the X or Z, respectively. However, the canonical model does not explicitly predict an expansion stage of sex-limited Y/W chromosomes during their evolution [5,14,15,16,17,18]. Such an expansion has been observed in plants [13] and fishes, amphibians, reptiles and some birds, many species of which have larger Ys and Ws than their Xs and Zs counterparts, suggesting that the accumulation of transposons and expansion of repetitive sequences can increase their size during their early differentiation [37,92]. This enlargement can be a rapid and effective mechanism to make the emerging Ys and Ws different from the other chromosomes and further prevent crossovers between the sex chromosomes. The shrinking of the Y and W chromosome to their familiar small size in mammals and birds is proposed to only occur at a later phase of their degeneration [37]. The enlarged Y/W chromosomes of some species are thus considered “younger” than the shrunk Y/W chromosomes of other species. Phylogenetic analysis based on current datasets supports this as the enlarged Y/W are “younger” than the shrunk ones in *Gastrotheca* frogs (Figure S2). However, the overall pattern in the whole phylogeny could also be explained by lineage-specific selection on the rate of sex chromosome differentiation (Section 5.1), and more data on such heteromorphic sex chromosome systems in the future will help resolve this issue. As large Ys/Ws lose genetic material to turn to small, they are expected to pass through a stage where they are identical in size to the homologous Xs/Zs chromosomes [13,37]. This hypothesis has gained support from genomic analysis of fish species with various stages of early evolution of their sex chromosomes [93]. Whether repetitive sequences are mainly responsible for the enlargement of Ys/Ws in anurans, as well as whether homomorphic sex chromosomes have passed through an enlarged stage of Ys/Ws in their past evolutionary history, is not clear for most frog species and would require comparative genomic analysis.

### 5.1. What Forces Result in Heteromorphic Sex Chromosomes in Frog Lineages?

The reasons why ~25% of studied frogs have escaped the mechanisms that seem to maintain homomorphic sex chromosomes in other species (“fountain of youth” and/or “hot-potato model”) remain unclear. Homomorphy has been maintained for over 100 Myr in many frog species (Figure S3). The old age of heteromorphic sex chromosome systems is also not a good explanation because phylogenetic analysis shows them to be as old as ~150 Mya and as recent as ~10 Mya (Figure S3).

Of particular interest are the frog lineages *Eleutherodactylus*, *Gastrotheca*, *Pristimantis*, *Engystomops*, which have primarily heteromorphic sex chromosomes (Figure S3, Table S1). They are all distributed in highly restricted ranges in the neotropics (central and south America) and many of the species occur in single Caribbean islands (sometimes more than one species co-occur in one island), or in small populations [12,30]. Their small population sizes might result in lineage-specific fast degeneration as an outcome of the fixation of deleterious genetic variation. Another possibility is lineage-specific selection on the rate of sex chromosome differentiation, a situation found in palaeognathous and neognathous birds in which limited divergence between sex chromosomes (i.e., sex chromosome are homomorphic and largely recombining) were detected spanning >90 Myr, suggesting slow rates of sex chromosome degeneration compared to the rest of bird lineages with highly degenerated sex chromosomes [24,94,95,96]. Unlike these primarily non-flying birds, these frog lineages might show faster degeneration, as observed in the heteromorphic sex chromosomes of the salamander genus *Aneides*, which are restricted to isolated populations [30]. Most frogs with homomorphic sex chromosomes are widely distributed in the mainland, such as in the lineages of *Rana*, *Hyla,* and *Bufo* frogs [30]. In addition, the frog lineages with primarily heteromorphic sex chromosomes (*Eleutherodactylus*, *Gastrotheca*, *Pristimantis*, *Engystomops*) have unique life-history traits that might contribute to fast sex chromosome degeneration. For instance, all *Eleutherodactylus* and *Pristimantis* frogs have direct development, i.e., they lack free-living aquatic tadpoles and instead hatch from terrestrial eggs as miniature adults [97,98,99,100]. They experience a reduced developmental time to reach adulthood, which leads to shorter generation times and could possibly accelerate sex chromosome evolution. These frogs, together with *Gastrotheca* frogs, also have parental care, and some show pronounced sexual dimorphism in body color and size [100]. *Engystomops* frogs have a foam-nest behavior, where the egg jelly is beaten into white foam by the male during amplexus, which functions as parental care. The developing eggs in the foam nest are removed from the aquatic environment and are protected from desiccation and predators [100]. These life history traits might lead to pronounced sexual selection compared to other frog lineages, which could generate strong selection for sexually antagonistic genes, making them more central in sex chromosome evolution, including their degeneration, in these lineages.

Other frogs with heteromorphic sex chromosomes occur in linages with a majority of species with homomorphic sex chromosomes (Figure 4). In these cases, it is more difficult to argue for demographic and life history explanations that allow the escape from the forces normally maintaining homomorphic sex chromosomes. One possibility that can allow the canonical model of sex chromosome evolution to hold despite male-specific absence of recombination (see Section 7) is the occurrence of a chromosomal inversion involving the sex-determining region, so that recombination arrest depends on genotypic, not phenotypic sex. For example, XY sex-reversed individuals would not recombine if the Y was fixed for an inversion. This is particularly relevant for W chromosome evolution. Since female-specific telomere-specific recombination does not seem to occur in frogs, the most likely mechanism of recombination restriction is similar to the mechanisms familiar from the canonical model of sex chromosome evolution. One case of recombination restriction is known (in *Buergeria buergeri*) where the ZW bivalent is ring-shaped, although the mechanism is unclear [101]. More comparative studies of sexual dimorphism and genomic composition across species with both homomorphic and heteromorphic sex chromosomes are needed to understand the different evolutionary trajectories of their sex chromosomes.

## 6. Sex Chromosome–Autosome Fusion

Fusions between the sex chromosomes and autosomes have been described for 11 frog species (Figure 1, Table S1). All are Y-autosome fusions in species with male heterogametic (XX/XY) sex chromosome systems. The higher fusion rate in the XX/XY system (11/94, compared to 0/45 in ZZ/ZW species) in frogs should be considered preliminary and taken with caution because the sample size is small. Nevertheless, it is consistent with the higher rate of fusions of Y chromosomes with autosomes, compared to X, Z or W chromosomes, which were observed in fishes and squamate reptiles [102]. Population genetic models suggest that direct selection acting on fusions or sexually antagonistic selection cannot alone account for the predominance of Y-autosome fusions. Instead, the most plausible explanations are that fusions are slightly deleterious, and the mutation rate is probably male-biased, or the reproductive sex ratio is female-biased [102].

## 7. Extremely Sexual Dimorphic Recombination Pattern

Many species show heterochiasmy, in which the location and rate of recombination differ between the sexes [103,104,105,106]. The most extreme case is achiasmy (the absence of recombination in one sex), which always occurs in the heterogametic sex (e.g., XY males in *Drosophila*; ZW females in butterflies) and has evolved at least 29–34 times independently in animals [103]. Sexual dimorphism in recombination may be a byproduct of mechanistic differences between meiosis in males and females, or it may be adaptive and selected to promote tight linkage of beneficial alleles on the Y or W [104,106,107,108]. Neither explanation is adequate for all species [106]. Furthermore, sex differences in recombination can vary in degree and direction even between closely related species [106,107,108].

**Figure 5:**
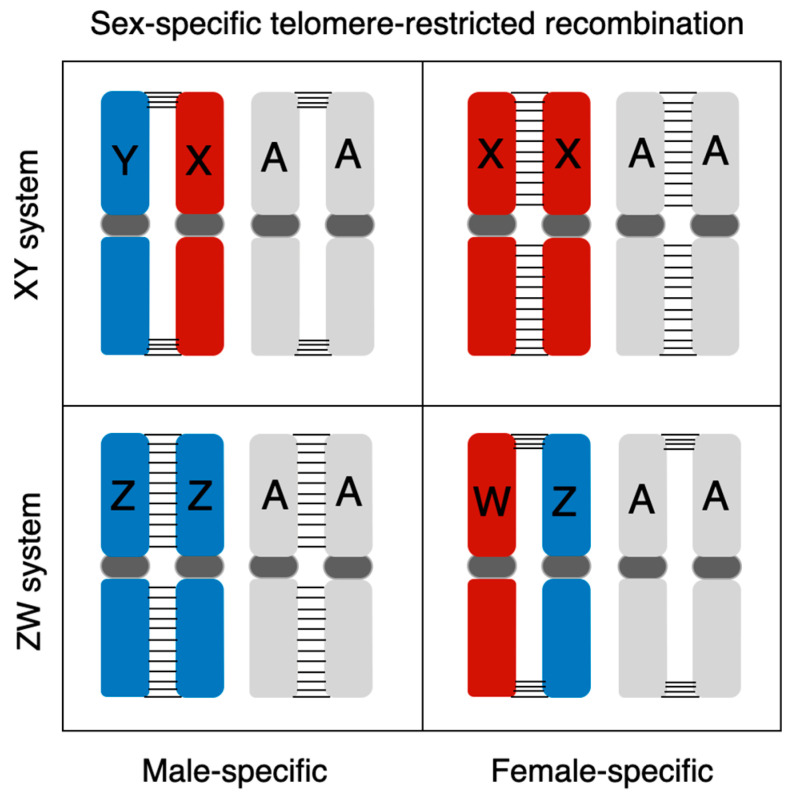
Schematic drawing of sex-specific telomere-restricted recombination in male heterogametic (XX/XY) and female heterogametic (ZZ/ZW) sex chromosome systems. “A” refers to all autosomes, and horizontal bar “-“ indicates recombination between homologous chromosomes. Note that female-specific telomere-restricted recombination has not been described in studied anurans. The only case of telomere-specific ZW recombination (in *Buergeria buergeri*) does not affect the autosomes.

Cytogenetic studies of frogs show that during male meiosis, ring-shaped bivalents form in most frog lineages with male heterogametic (XX/XY) sex chromosomes (male-specific telomere-restricted recombination), suggesting that most recombination occurs in the telomere regions in males but across the chromosome length in females (Figure 5, [12,30]). Extreme heterochiasmy has been confirmed by directly estimating sex-specific recombination with genetic mapping in many frogs and some poecilid fishes with XY systems [20,43,109,110], as well as in a few ZW systems in frogs [25,111], where males always show reduced recombination. However, all of these studies involve frogs with homomorphic sex chromosomes. Cytogenetic analysis of female meiosis in the Kajika frog (*Buergeria buergeri*), which has a homomorphic ZW system, showed the ZW bivalent to be ring-shaped, while the autosomes showed recombination across their length, suggesting that female-specific telomere-restricted recombination has not evolved in this case (Figure 5, [30,107]). Little is known about female-specific telomere-restricted recombination in other female heterogametic (ZZ/ZW) frog species, especially in those with heteromorphic sex chromosomes, which has the potential to affect evolution of the W chromosome in the same way that male-specific telomere-restricted recombination influences Y evolution in most studied frogs. The possible evolution of female-specific telomere-restricted recombination (Figure 5), or chromosome-specific (i.e., ZW) recombination suppression, can both affect the evolution of ZW systems, and future comparative studies of homomorphic and heteromorphic ZW systems are needed to understand their relative importance in sex-chromosome evolution.

## Data Availability

All datasets in this review have been provided in Supplementary Table S1.

## Supplementary Materials

The following are available online at https://www.mdpi.com/article/10.3390/genes12040483/s1, Table S1. Characterized sex chromosome systems in Anura frogs from published literature. The columns show species, chromosome number, sex chromosome morphology, sex-determination system, references and other relevant notes. Figure S1. Distribution of various sex-determination systems along Anurans. The phylogenetic tree with estimated divergence time is obtained from http://www.timetree.org/, accessed on 5 February 2021. Pie charts display the posterior probability of sex-determination systems on each node of the phylogenetic tree. Figure S2. Distribution of homomorphic and various heteromorphic sex chromosome systems (i.e., Y/W bigger than X/Z, Y/W smaller than X/Z, other forms) along the phylogenetic relationship in the studied anurans. The phylogenetic tree with estimated divergence time is obtained from http://www.timetree.org/, accessed on 5 February 2021. Pie charts display the posterior probability of sex chromosome morphology on each node of the phylogenetic tree. Figure S3. Distribution of homomorphic and heteromorphic sex chromosomes along the phylogenetic relationship in the studied anurans. Homomorphic sex chromosomes are indicated in orange, and heteromorphic in purple. The phylogenetic tree with estimated divergence time is obtained from http://www.timetree.org/, accessed on 5 February 2021. Pie charts display the posterior probability of sex chromosome morphology on each node of the phylogenetic tree.
