# Supplementary material for: The Diversity and Evolution of Sex Chromosomes in Frogs
Source: Genes (Basel). 2021 Mar 26;12(4):483. doi: 10.3390/genes12040483 (PMC8067296; doi:10.3390/genes12040483)
Supplement: Supplementary file 1 [file genes-12-00483-s001.zip › suppl/FigureS1.pdf]

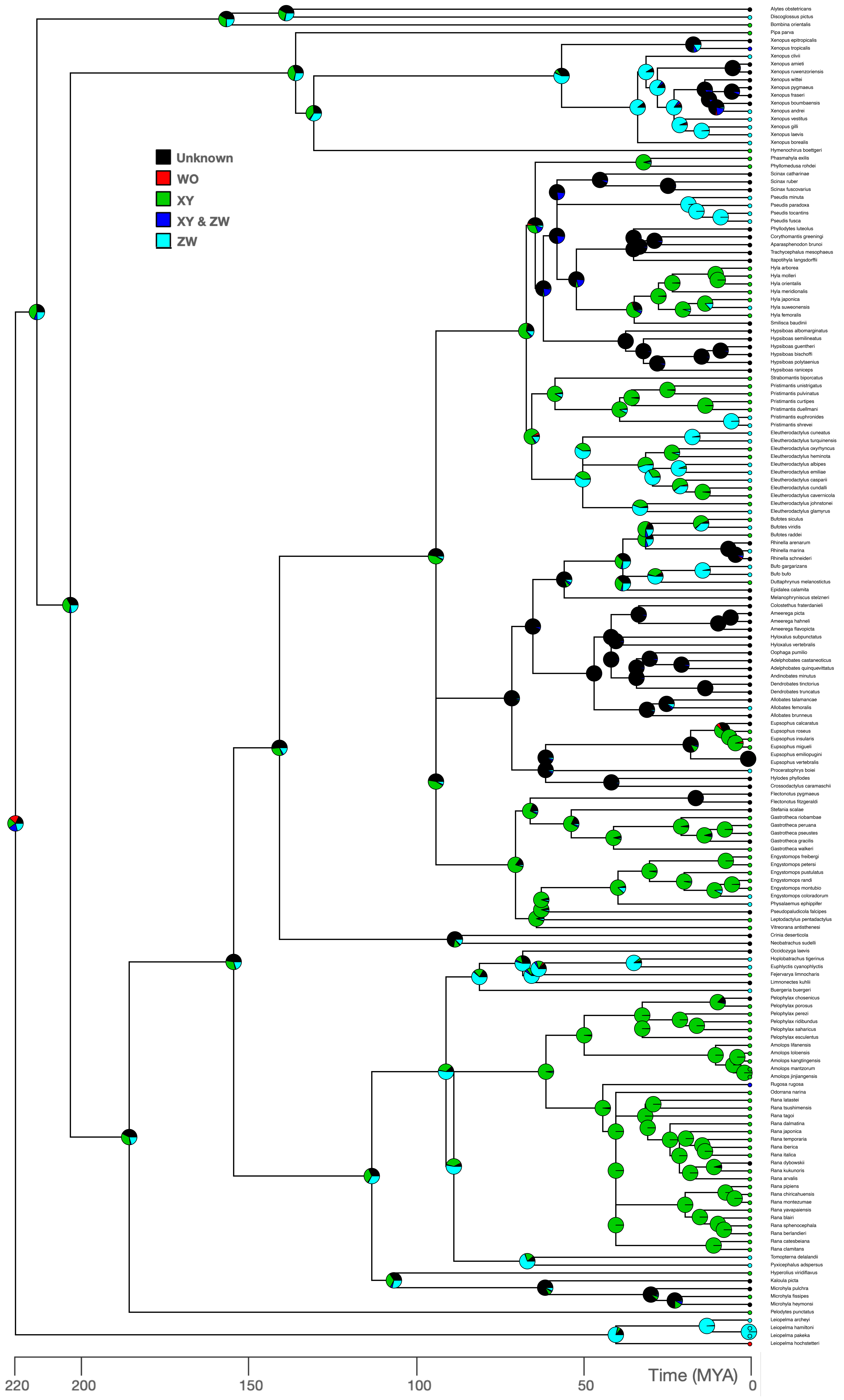
Unknown
WO
XY
XY & ZW
ZW
Alytes obstetricans
Discoglossus pictus
Bombina orientalis
Pipa parva
Xenopus epitropicalis
Xenopus tropicalis
Xenopus clivii
Xenopus amieti
Xenopus ruwenzoriensis
Xenopus wittei
Xenopus pygmaeus
Xenopus fraseri
Xenopus boumbaensis
Xenopus andrei
Xenopus vestitus
Xenopus gilli
Xenopus laevis
Xenopus borealis
Hymenochirus boettgeri
Phasmahyla exilis
Phyllomedusa rohdei
Scinax catharinae
Scinax ruber
Scinax fuscovarius
Pseudis minuta
Pseudis paradoxa
Pseudis tocantins
Pseudis fusca
Phyllodytes luteolus
Corythomantis greeningi
Aparasphenodon brunoi
Trachycephalus mesophaeus
Itapotihyla langsdorffii
Hyla arborea
Hyla molleri
Hyla orientalis
Hyla meridionalis
Hyla japonica
Hyla suweonensis
Hyla femoralis
Smilisca baudinii
Hypsiboas albomarginatus
Hypsiboas semilineatus
Hypsiboas guentheri
Hypsiboas bischoffi
Hypsiboas polytaenius
Hypsiboas raniceps
Strabomantis biporcatus
Pristimantis unistrigatus
Pristimantis pulvinatus
Pristimantis curtipes
Pristimantis duellmani
Pristimantis euphronides
Pristimantis shrevei
Eleutherodactylus cuneatus
Eleutherodactylus turquinensis
Eleutherodactylus oxyrhyncus
Eleutherodactylus heminota
Eleutherodactylus albipes
Eleutherodactylus emiliae
Eleutherodactylus casparii
Eleutherodactylus cundalli
Eleutherodactylus cavernicola
Eleutherodactylus johnstonei
Eleutherodactylus glamyrus
Bufotes siculus
Bufotes viridis
Bufotes raddei
Rhinella arenarum
Rhinella marina
Rhinella schneideri
Bufo gargarizans
Bufo bufo
Duttaphrynus melanostictus
Epidalea calamita
Melanophryniscus stelzneri
Colostethus fraterdanieli
Ameerega picta
Ameerega hahneli
Ameerega flavopicta
Hyloxalus subpunctatus
Hyloxalus vertebralis
Oophaga pumilio
Adelphobates castaneoticus
Adelphobates quinquevittatus
Andinobates minutus
Dendrobates tinctorius
Dendrobates truncatus
Allobates talamancae
Allobates femoralis
Allobates brunneus
Eupsophus calcaratus
Eupsophus roseus
Eupsophus insularis
Eupsophus migueli
Eupsophus emiliopugini
Eupsophus vertebralis
Proceratophrys boiei
Hylodes phyllodes
Crossodactylus caramaschii
Flectonotus pygmaeus
Flectonotus fitzgeraldi
Stefania scalae
Gastrotheca riobambae
Gastrotheca peruana
Gastrotheca pseustes
Gastrotheca gracilis
Gastrotheca walkeri
Engystomops freibergi
Engystomops petersi
Engystomops pustulatus
Engystomops randi
Engystomops montubio
Engystomops coloradorum
Physalaemus ephippifer
Pseudopaludicola falcipes
Leptodactylus pentadactylus
Vitreorana antisthenesi
Crinia deserticola
Neobatrachus sudelli
Occidozyga laevis
Hoplobatrachus tigerinus
Euphlyctis cyanophlyctis
Fejervarya limnocharis
Limnonectes kuhlii
Buergeria buergeri
Pelophylax chosenicus
Pelophylax porosus
Pelophylax perezi
Pelophylax ridibundus
Pelophylax saharicus
Pelophylax esculentus
Amolops lifanensis
Amolops loloensis
Amolops kangtingensis
Amolops mantzorum
Amolops jinjiangensis
Rugosa rugosa
Odorrana narina
Rana latastei
Rana tsushimensis
Rana tagoi
Rana dalmatina
Rana japonica
Rana temporaria
Rana iberica
Rana italica
Rana dybowskii
Rana kukunoris
Rana arvalis
Rana pipiens
Rana chiricahuensis
Rana montezumae
Rana yavapaiensis
Rana blairi
Rana sphenocephala
Rana berlandieri
Rana catesbeiana
Rana clamitans
Tomopterna delalandii
Pyxicephalus adspersus
Hyperolius viridiflavus
Kaloula picta
Microhyla pulchra
Microhyla fissipes
Microhyla heymonsi
Pelodytes punctatus
Leiopelma archeyi
Leiopelma hamiltoni
Leiopelma pakeka
Leiopelma hochstetteri
220
200
150
100
50
Time (MYA)
0
