# Supplementary figures and images for: The Diversity and Evolution of Sex Chromosomes in Frogs
Source: Genes (Basel). 2021 Mar 26;12(4):483. doi: 10.3390/genes12040483 (PMC8067296; doi:10.3390/genes12040483)

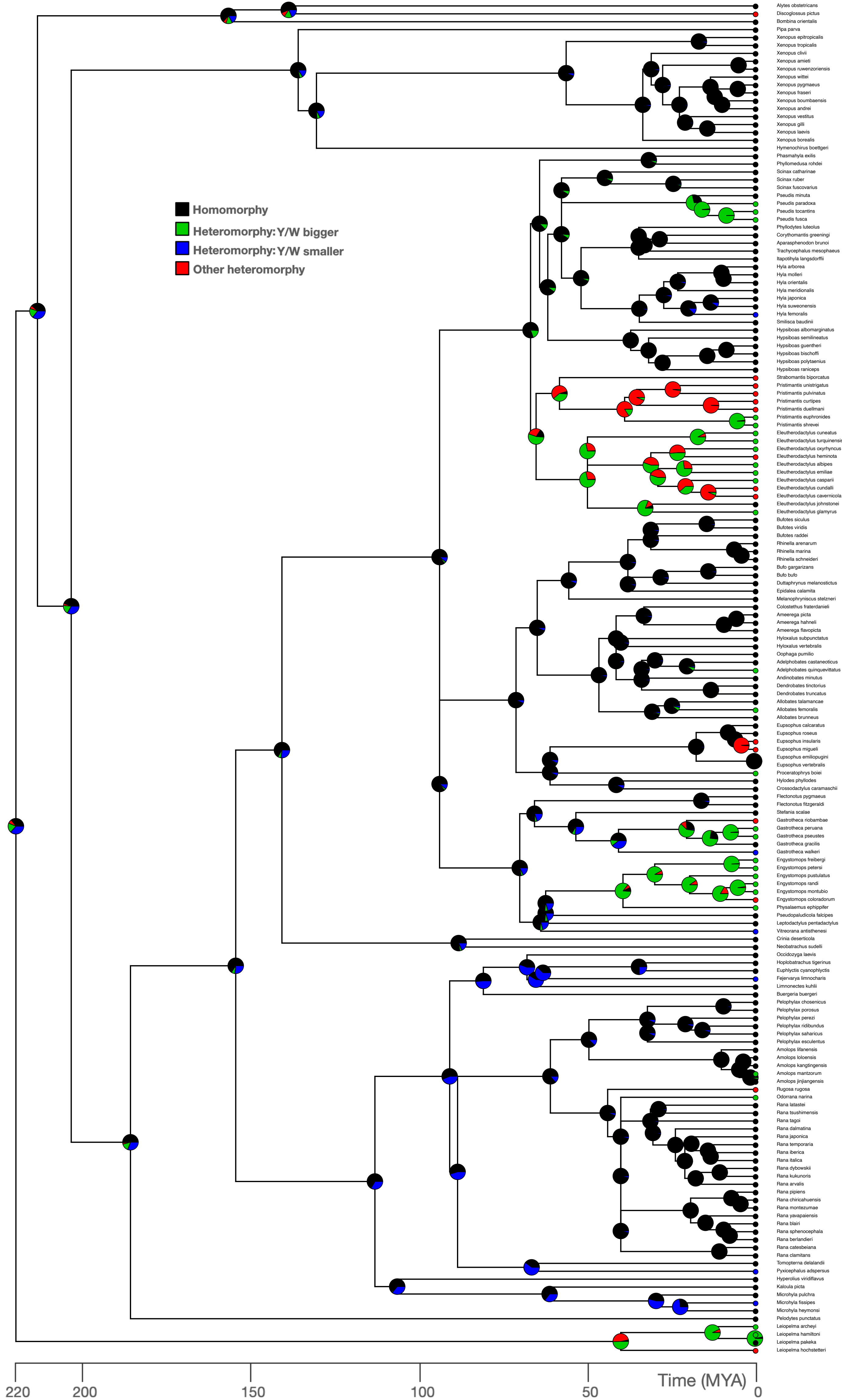

Supplement: Supplementary file 1 [file genes-12-00483-s001.zip › suppl/FigureS2.pdf]

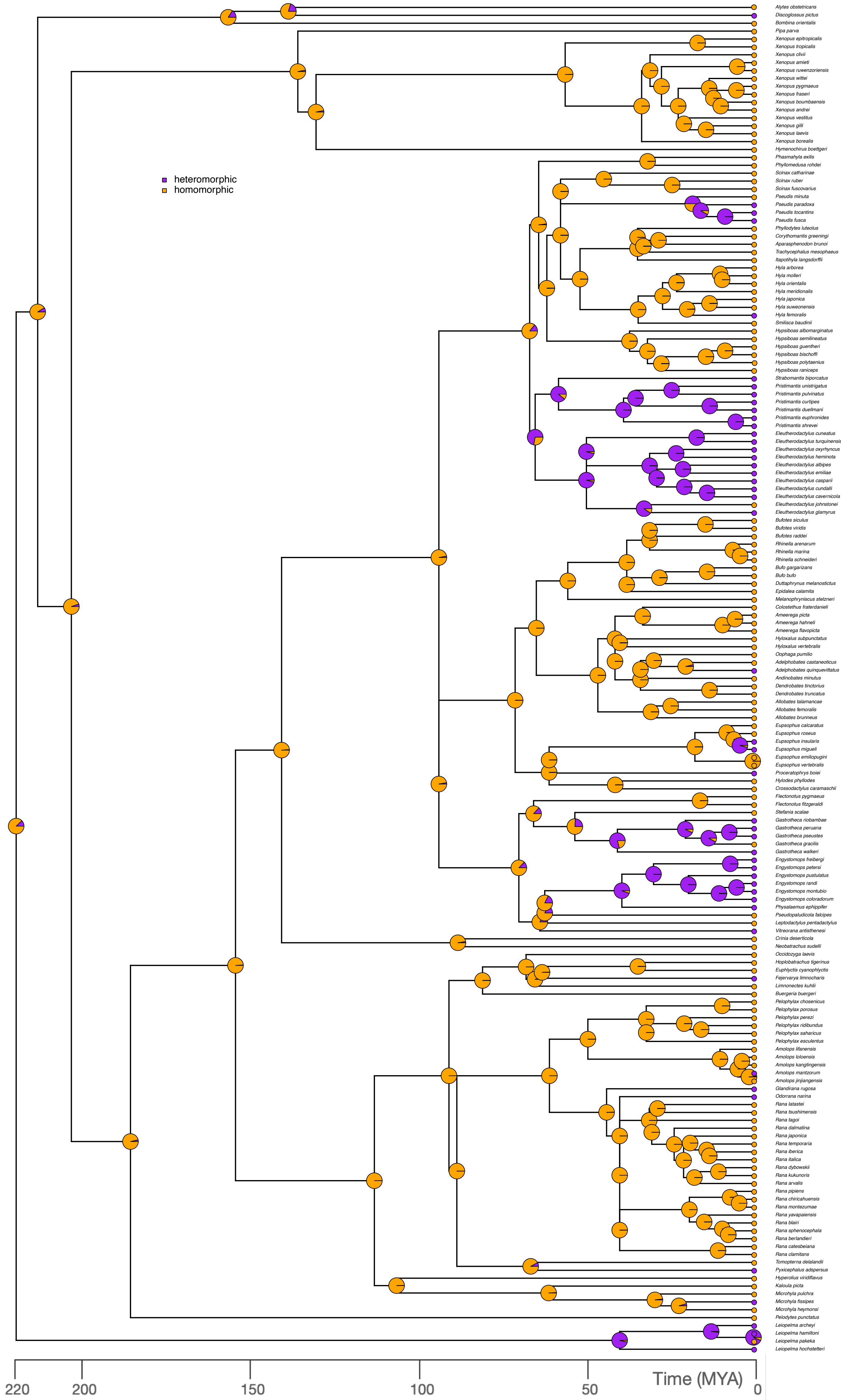

Supplement: Supplementary file 1 [file genes-12-00483-s001.zip › suppl/FigureS3.pdf]
